# Biomechanical stability of magnesium plate and screw fixation systems in LeFort I osteotomy: a three-dimensional finite element analysis

**DOI:** 10.1186/s40902-024-00451-8

**Published:** 2024-12-18

**Authors:** Su-Min Cho, Byoung-Eun Yang, Won-Hyeon Kim, Sang-Yoon Park, Sung-Woon On, Jong-Ho Lee, Soo-Hwan Byun

**Affiliations:** 1 Department of Prosthodontics, Hallym University Sacred Heart Hospital, Anyang, 14066 Republic of Korea; 2 Graduate School of Clinical Dentistry, Hallym University, Chuncheon, 24252 Republic of Korea; 3 Department of Oral and Maxillofacial Surgery, Hallym University Sacred Heart Hospital, Anyang, 14066 Republic of Korea; 4 Dental Life Science Research Institute/Innovation Research & Support Center for Dental Science, Seoul National University Dental Hospital, Seoul, 03080 Republic of Korea; 5 Institute of Clinical Dentistry, Hallym University, Chuncheon, 24252 Republic of Korea; 6 Department of Oral and Maxillofacial Surgery, Hallym University Dongtan Sacred Heart Hospital, Hwaseong, 18450 Republic of Korea; 7 Oral Oncology Clinic, Research Institute & Hospital, National Cancer Center, Goyang, 10408 Republic of Korea; 8 Dental Robotic Center, Hallym University Sacred Heart Hospital, Anyang, 14066 Republic of Korea

**Keywords:** Biodegradable, Magnesium plate, LeFort I osteotomy, Finite element analysis, Titanium plate

## Abstract

**Background:**

Titanium (Ti–6Al–4 V) is used for fixation in LeFort I osteotomy, a procedure for treating midface deformities. This study assessed the biomechanical stabilities of two Mg alloys (WE43 and ZK60) as biodegradable alternatives and compared them against Ti using finite element analyses. The LeFort I osteotomy procedure was simulated, and various plate and screw configurations were tested. The maximum principal and peak von Mises stresses in the metal plates and bone screws were measured under four load conditions, and the stability was evaluated.

**Results:**

The holes in the Mg screws, as compared with the Ti counterparts, exhibited higher and lower stress levels in the cortical and cancellous bones, respectively. The Mg screws also exhibited a higher fracture risk. The ZK60 plate, as compared with the Ti and WE43 plates, exhibited a lower fracture risk under all load conditions. ZK60 exhibited higher biomechanical stability in terms of maintaining the gap between osteotomy surfaces and lower fracture risk; the osteotomy surfaces with Ti im-plants underwent bone impaction, resulting in gap closure.

**Conclusions:**

Although the Mg implants exhibited better stress distribution, their screw strength requires improvement. Appropriate improvements can promote the use of Mg alloys in bone fixation applications.

## Background

LeFort I osteotomy is one of the most frequently used procedures for treating midface deformities and malocclusion [1]. This technique was introduced in 1859 by von Langenbeck, who described it as the osteoplastic resection of the maxilla. This method has evolved over time, with detailed documentation of numerous cases and variations in operative techniques [2]. In this procedure, the maxilla and palatine are separated by a horizontal bone cut that passes through the lower lateral part of the maxilla and lower one-third of the septum. Subsequently, the bone fragment is repositioned based on the preoperative reference point [3]. Upon the accurate repositioning of the lower bone fragments, the bone fragments must be secured with fixation. Two key fixation techniques are employed, namely, combined wire fixation and rigid internal fixation. Combined wire fixation involves wire osteosynthesis augmented by circumzygomatic suspension wires and maxillomandibular fixation, whereas rigid internal fixation involves the use of small-plate osteosynthesis to stabilize the LeFort I osteotomy. Rigid internal fixation offers enhanced stability, as compared with combined wire fixation [4]. Plate osteosynthesis has steadily advanced over the last 200 years to address the three key requirements of fracture management, namely, long-term fixation, preservation of biological integrity, and fracture healing promotion [5]. Several factors, including the position, size, number of plates, and gap size between the cut bones, influence the success of plate and screw fixation.

The Ti in Ti–6Al–4 V exhibits excellent bonding with bone and is used as the standard material for non-resorbable and permanent bone fixation. Titanium can resist the diversion of masticatory muscles induced by fracture fragments because it elicits a significantly lower aggressive reaction to foreign bodies. Additionally, Ti can withstand considerable loads before failing, thereby preserving anatomic alignment [6]. However, concerns owing to potential development restrictions, transcranial movement of internal hardware, and the eventual requirement for hardware removal in juvenile patients have arisen [6]. Moreover, the plate and screws in 10‒12% of surgical patients must be removed owing to discomfort, infection, or pain, necessitating secondary surgery [7].

Resorbable plates composed of polylactic acid, polyglycolic acid, polydioxanone acid, and Mg have been developed to address these challenges. Resorbable plates minimize growth constraints by maintaining sufficient strength until healing occurs; subsequently, they are resorbed via hydrolysis within 12–14 months. The most used resorbable materials in medical applications are poly-L-lactic acid (PLLA) and Mg. Notably, PLLA is a biocompatible synthetic polymer, whereas Mg is biodegradable. PLLA plates exhibit high mechanical strength, thereby providing stability and support during the healing process. However, PLLA plates are not as strong as metal plates. Moreover, they must be larger than metal plates to achieve comparable stability. In contrast, the mechanical properties, that is, stiffness and strength, of Mg plates are like those of natural bone. Additionally, biodegradable polymer-based bone fixation systems are translucent and radiolucent, which renders postoperative X-ray diagnosis and clinical differentiation between the plate and screw holes challenging [8]. Unlike permanent metals and resorbable polymers, degradable Mg alloys provide strength and radiopacity [9].

Although Mg has potential utility in medical applications, it produces hydrogen upon absorption [10, 11], and its strength has not been conclusively demonstrated The in vivo corrosion of Mg-based implants in biomaterial applications results in the generation of a soluble, nontoxic oxide, which is subsequently eliminated through urination [11]. Additionally, Mg can stimulate the formation of new bone tissue owing to its physiological properties and presence in bone tissue [12]. To be used as lightweight, biodegradable, and load-bearing implants, Mg and its alloys must remain within the body for 12‒18 weeks while the bone tissue heals. Moreover, Mg implants must be eventually replaced with natural tissue. Alloying is a universal method for enhancing the mechanical strength and corrosion resistance of Mg. Among the various alloying elements, Zn and Zr are generally used owing to their strengthening properties and high biocompatibility. In particular, Zn can enhance the corrosion resistance of Mg alloys by reducing the corrosive effects of impurities such as Fe and Ni [13]. In contrast, Zr is typically used as a grain refiner to enhance the strength of Al alloys and mitigate the adverse effects of Fe contamination on corrosion resistance [14].

WE-type alloys (Mg–Y–Re–Zr) were previously evaluated in vitro and in vivo as potential materials for orthopedic applications. WE-type alloys, as compared with other Mg alloys, exhibit superior corrosion resistance and cytocompatibility [12]. The biocompatibility of ZK60 and WE-type alloys is comparable to that of hydroxyapatite [15]. Therefore, ZK60 and WE43 Mg alloys are biocompatible and exhibit higher mechanical strength than pure Mg.

Finite element analysis (FEA) is a computational method for modeling the mechanical behavior of complex structures for which analytical equations are inadequate. FEA is useful in various applications, including medical scenarios such as the assessment of facial fractures and plating techniques. The equations can be solved to provide insights into stress and strain distributions. In particular, FEA is a powerful tool for understanding complex mechanical behaviors and making informed decisions in engineering and medicine [16]. Although several studies focused on the FEA of Ti and polymers, related research on Mg remains scarce [17–19].

This study aimed to evaluate the biomechanical stability of two Mg alloys (WE43 and ZK60) using FEA. These alloys were compared with Ti in the context of LeFort I osteotomy. Models based on the three materials were established for the same metal plate and screw shape, followed by FEA to investigate craniomaxillofacial fixation.

## Methods

### Bone shape and composition of surgical model

**Fig. 1:**
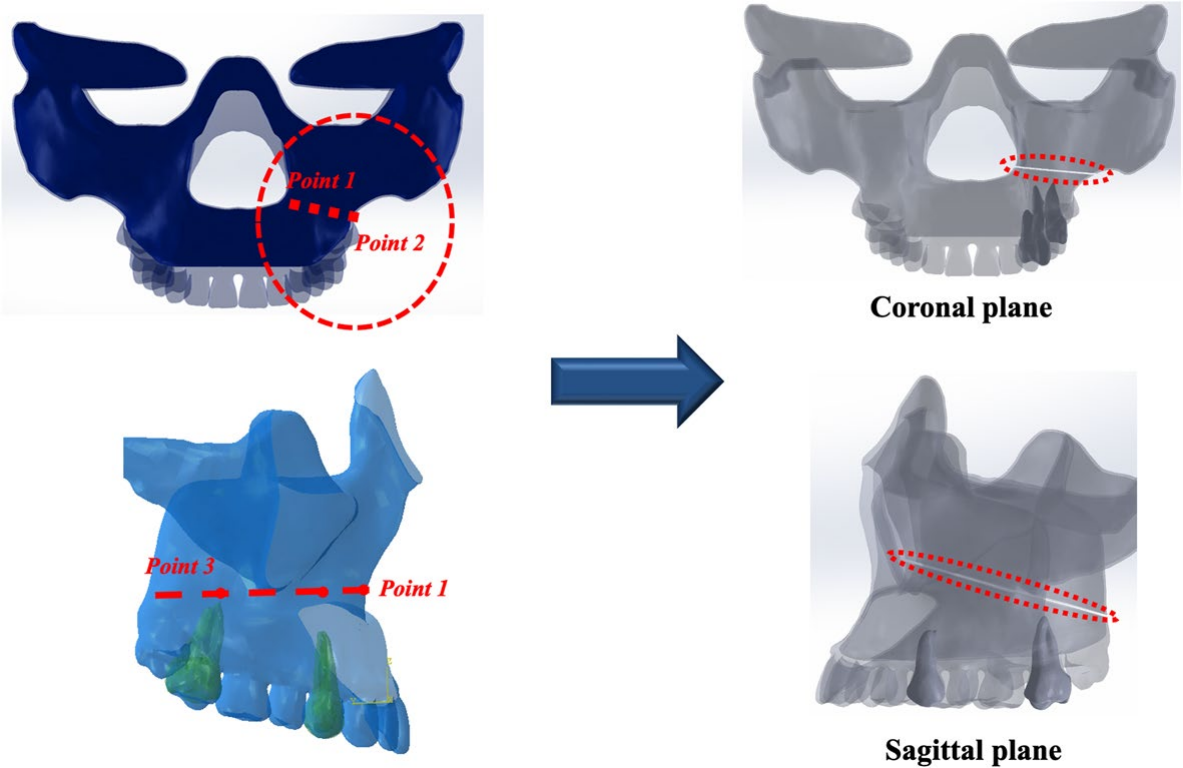
LeFort I osteotomy fracture model

The plate and screw system were designed using three-dimensional (3D) computer-aided design (CAD) modeling. To compare the biomechanical stability of Ti and the Mg alloys, a cone-beam computed tomography image of a standard adult skull was converted to a cross-sectional image using Mimics version 19.1 (Materialise Inc., Louvain, Belgium), which was then used to reconstruct the 3D facial bone model shape (SolidWorks 2016, Dassault Systems, Velizy, France). Cortical and cancellous bones were differentiated, and the cortical bone was set to have a constant thickness of 0.5 mm. Subsequently, two points in the coronal plane and a third point in the sagittal plane were defined as the horizontal bone-cutting plane passing through the inferior lateral region of the maxilla and inferior lower third of the septum to simulate the LeFort 1 osteotomy (Fig. 1). The osteotomy surface was defined using points to separate the maxilla from the palatine, and an osteotomy gap of 0.5 mm was established. This was used to construct the CAD-based osteotomy model (ABAQUS 2016, Dassault Systems, Velizy, France).

**Fig. 2:**
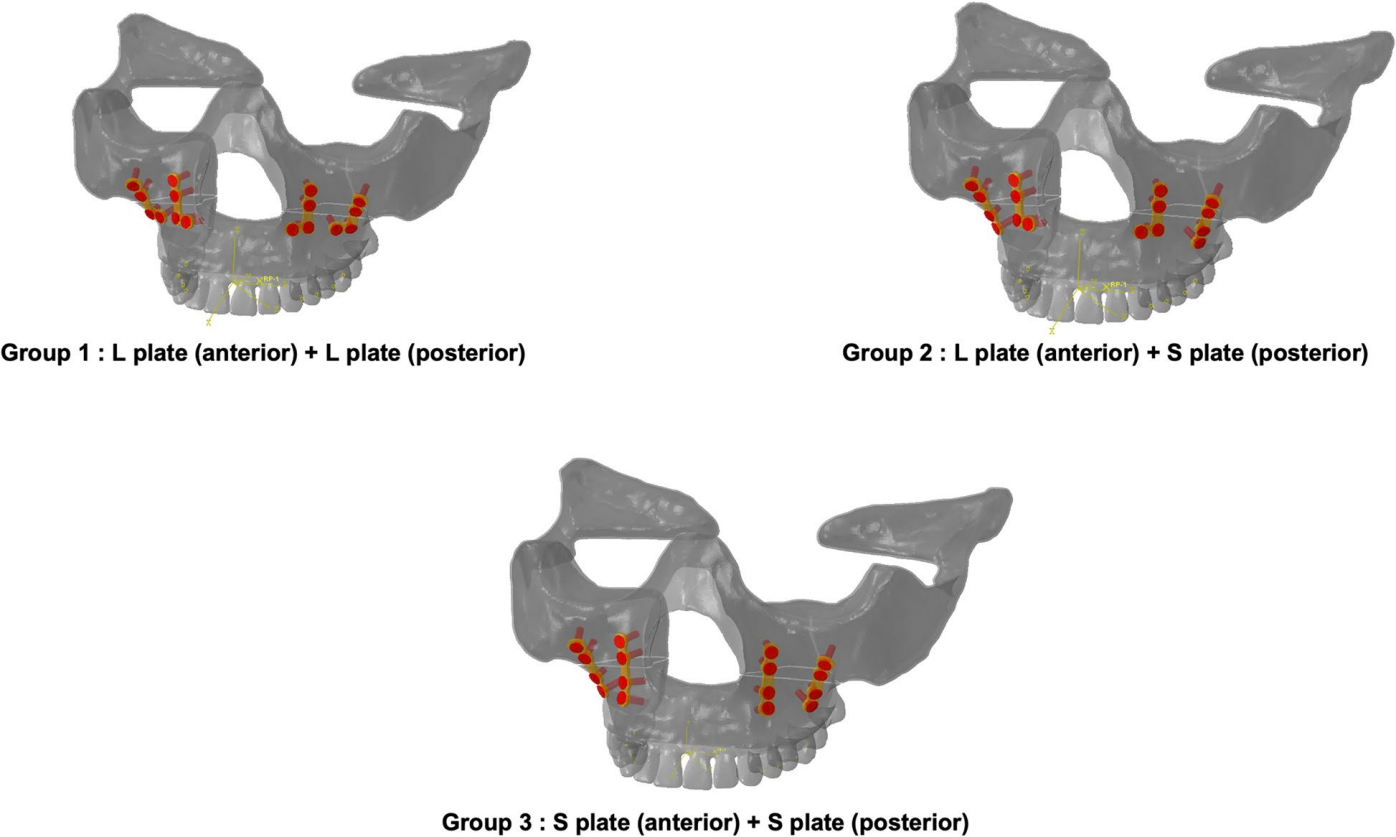
Plate types in the LeFort I osteotomy model

A customized metal plate was fabricated on the surface of the maxillary cortical bone using the LeFort I osteotomy model. Notably, patient-specific reconstruction plates can be generated using additive manufacturing technologies and computer-guided surgical planning [20]. The plates were categorized into three groups based on their shapes and combinations, namely, Group 1 consisted of L-shaped plates (L-plate) in the anterior and posterior parts; Group 2 consisted of a L-plate in the anterior part and a straight plate (S-plate) in the posterior part, and Group 3 comprised S-plates in the anterior and posterior parts (Fig. 2).

**Fig. 3:**
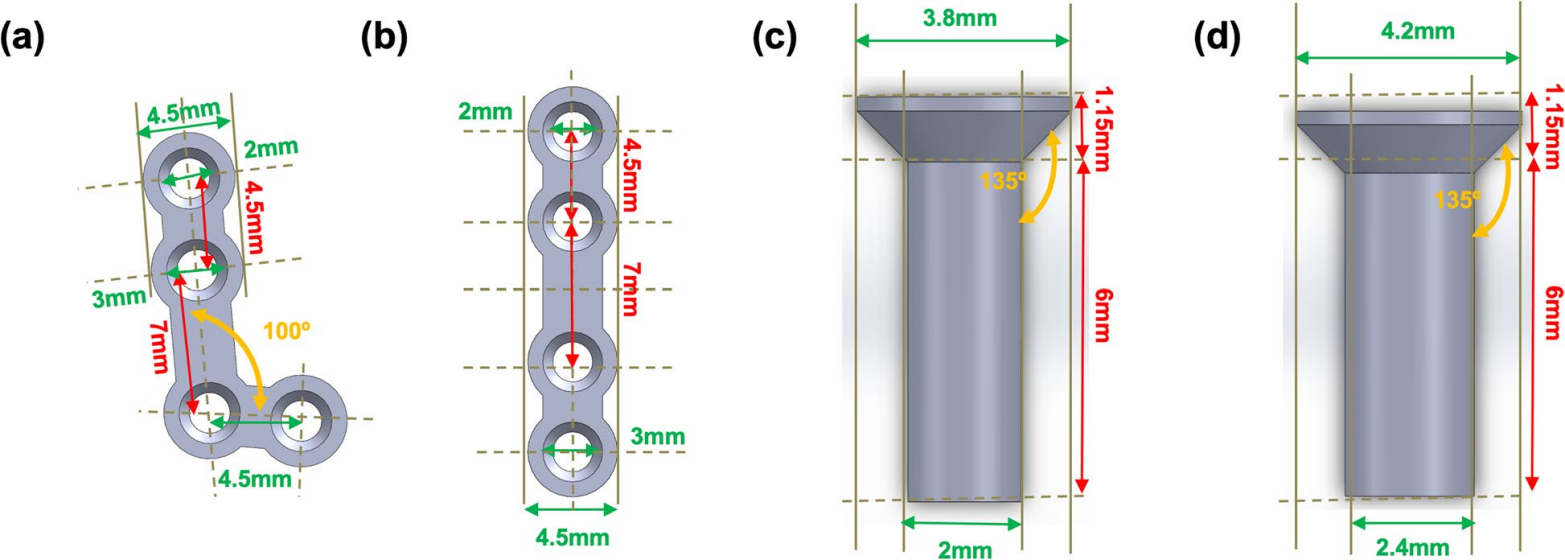
Types of Ti and Mg resorbable plates: **a** L-plate, **b** S-plate, **c** Ti screw, and **d** Mg screw. The screw specifications for the plates are also shown

Each treatment model incorporated three types of materials, namely, Ti, WE43, and ZK60, and was divided into nine subgroups. The thicknesses of the Ti and Mg plates were 0.4 and 1.0 mm, respectively. Sixteen screws were used, with 4 screws affixed per plate. The screw diameters differed slightly owing to the variations in plate thickness. The overall length of the Ti and Mg screws was the same. However, the Mg screws had body and head diameters of 2.4 and 4.2 mm, respectively, each dimension being 0.4 mm larger than those of the Ti screws (Fig. 3) [21]. Moreover, the Mg-alloy plates were designed slightly larger and thicker than the Ti plates because of their inherent material characteristics (Table 1).

**Table 1:** Physical properties of the materials used in this study

Material	Young’s modulus (MPa)	Poisson's ratio	Yield strength (MPa)
Cortical bone	15,000	0.330	-
Cancellous bone	1500	0.300	-
Titanium	113,800	0.342	880
WE43	44,000	0.270	198
ZK60	45,000	0.350	305

### Loads, boundaries, and contact conditions

**Fig. 4:**
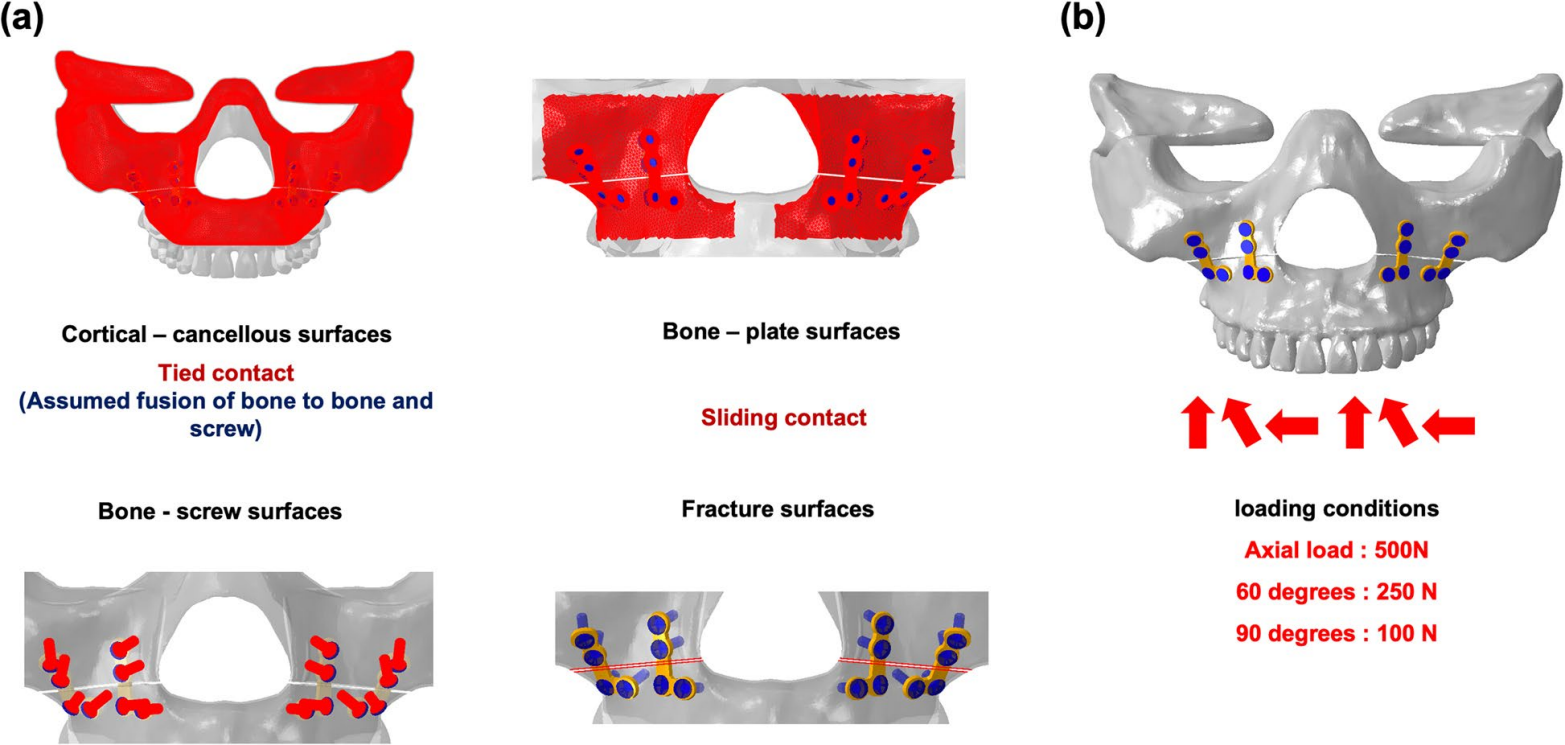
Detailed conditions for the finite element models: **a** contact and **b** loading conditions

A tie-contact boundary condition was applied in the analytical model to fully constrain the lower end of the cortical and cancellous bones, preventing movement and rotation in all directions. The same condition, corresponding to a fusion-type condition, was applied between the screw and bone and the screw and metal plate. The sliding-contact boundary condition was applied between the bone and plate surface, with the friction coefficient set as 0.3. A contact condition without a friction coefficient was applied between the osteotomy surfaces (Fig. 4).

To account for the load induced by various angles in the oral cavity, three load conditions, namely, vertical, 60°, and 90°, were designed with reference to the literature [1, 3, 22]. The magnitudes of the vertical load were 100 and 500 N. Additionally, the 60 and 90° loads were 250 and 100 N, respectively [23–26]. These loads were distributed across eight points, namely, four on the left and four on the right, and the applied load on each point was identical.

### Variables

The maximum principal stress (MPS), peak von Mises stress (PVMS), and gap between the bone fragments were derived for the metal plate and bone screws. The MPS was used to predict bone fracture, whereas PVMS was used to predict the failure of the plates and screws. The failure risk was defined as the ratio between the PVMS and yield strength, with deformation being indicated by a value exceeding 100%. Stress values for all three groups were obtained. However, results related to the screw hole locations for the cortical bone were obtained only when Group 1 plates composed of Ti and WE43 were used to control for other variables.

## Results

### Stress of the screw hole on the maxilla

**Fig. 5:**
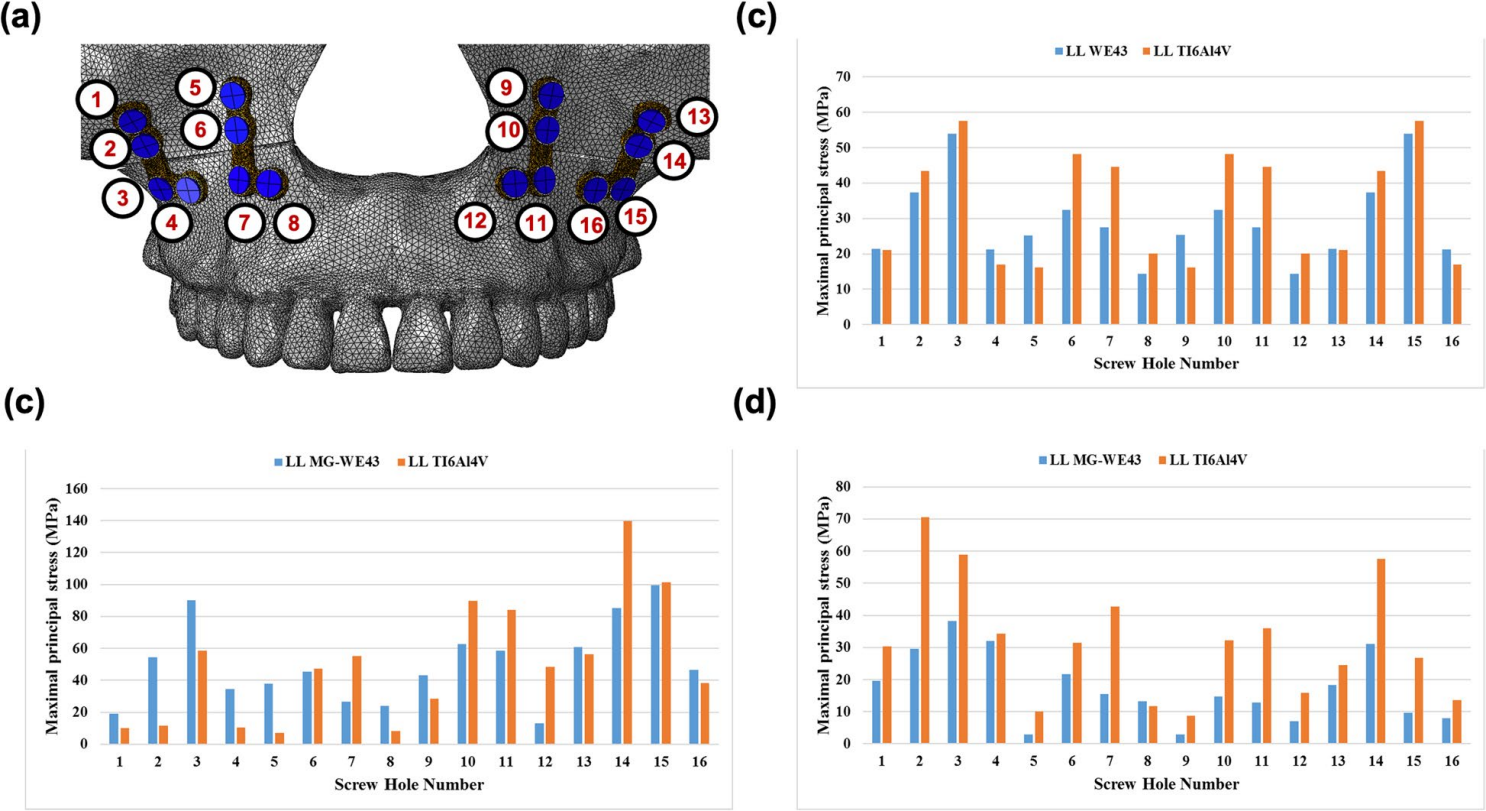
Maximum principal stress (MPS) of the screw hole in the cortical bone according to loading conditions: **a** screw and its hole number, **b** axial load (100 N), **c** 60° (250 N), and **d** 90° (100 N)

**Fig. 6:**
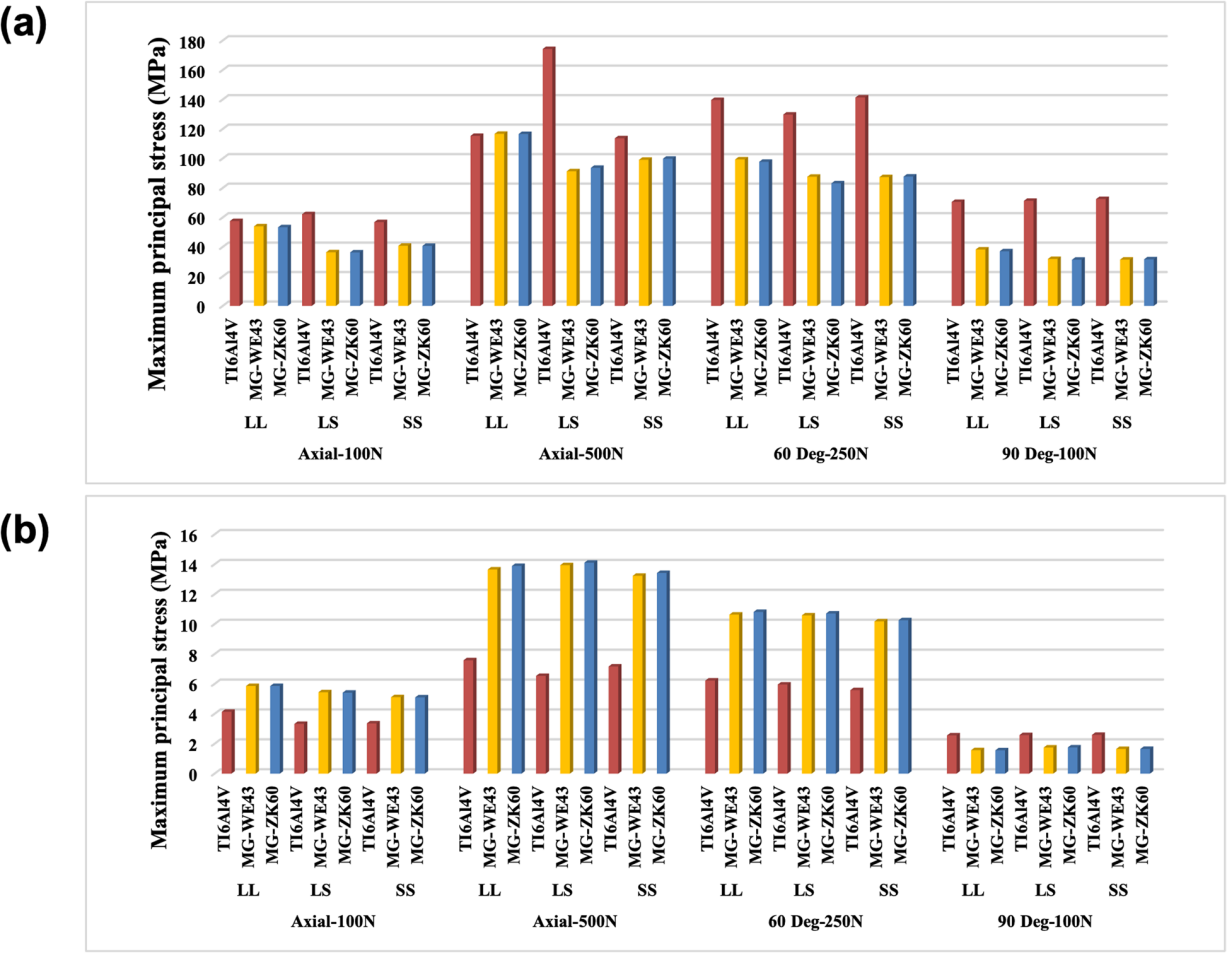
MPS (MPa) in the screw hole of the maxilla: **a** cortical and **b** cancellous bones

The screw holes closer to the osteotomy line exhibited higher stresses (Fig. 5). Despite its proximity to the osteotomy line, the area corresponding to the canine buttress did not exhibit stress. This trend was consistent across all groups. The MPS was concentrated in the cortical bone under all load conditions (Fig. 6). Excluding the axial load of 500 N in Group 1, the Mg alloys in all groups exhibited lower stress in the cortical bone and higher stress in the cancellous bone than those of the Ti alloy. The maximum stress value in the cortical bone using Ti materials was higher than when using Mg materials.

### Bone screw stress

**Fig. 7:**
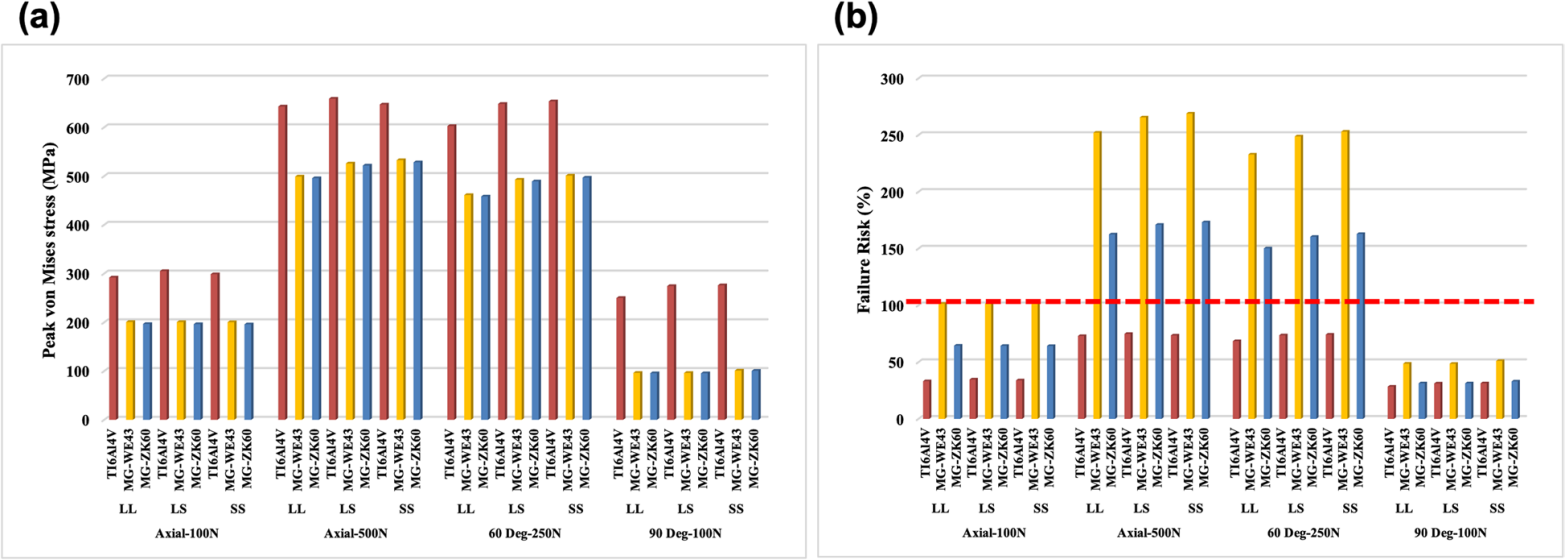
**a** Peak von Mises stress (PVMS; MPa) and **b** failure risk of the bone screw

Significant stresses were observed in screws closer to the osteotomy line; this trend was observed in the screw holes across all groups. The peak von Mises stress (PVMS) for Mg indicates that the fracture risk at a force > 250 N exceeded 100%. In contrast, the Ti material was predicted to be stable under all load conditions (Fig. 7). The fracture risk for ZK60 was lower than that for WE43 in all groups.

### Bone plate stress

**Fig. 8:**
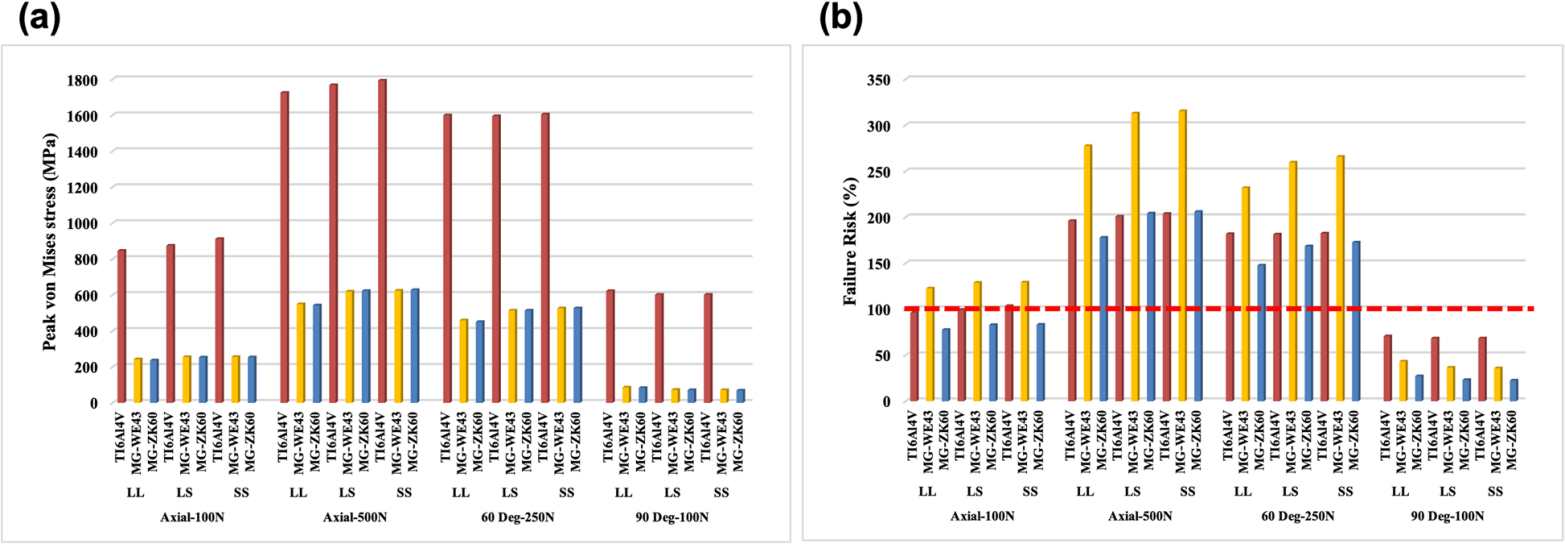
**a** PVMS (MPa) and **b** failure risk of the bone plate

The PVMS distribution patterns were similar for different materials but differed based on the position of each metal plate. In particular, the Ti metal plate exhibited significantly higher stress than the Mg plate (Fig. 8). This suggests that stress was highly concentrated on the Ti metal plate, and the stress distribution between the bone screw and Mg metal plate was more uniform. Excluding the horizontal load (90°, 100 N), WE43 exhibited a high fracture risk under all load conditions. Titanium and ZK60 exhibited low fracture risks only under the 100 N load (vertical and horizontal cases). The fracture risk of ZK60 was lower than that of Ti.

### Gap distance

Gap closing occurred in the Ti group under the 100-N axial load condition, where the upper and lower fragments came into contact. Moreover, the gap angle was approximately 1.6-fold larger than that of the Mg group. The 90°–100 N condition, wherein the load was mainly applied laterally, yielded no significant alterations in the gap or differences between the materials (Table 2).

**Table 2:** Gap distances under 100 N axial and 90°–100 N load conditions

Surgical model		Axial-100 N		90°-100 N	
		**Gap distance (mm)**	**Gap angle (°)**	**Gap distance (mm)**	**Gap angle (°)**
LL	Ti–6Al–4 V	Closing	0.761	0.512	0.017
	Mg-WE43	0.203	0.474	0.487	0.017
	Mg-ZK60	0.203	0.474	0.487	0.017
LS	Ti–6Al–4 V	Closing	0.759	0.512	0.0171
	Mg-WE43	0.23	0.433	0.488	0.017
	Mg-ZK60	0.23	0.433	0.488	0.017
SS	Ti–6Al–4 V	Closing	0.758	0.492	0.0123
	Mg-WE43	0.232	0.425	0.486	0.0172
	Mg-ZK60	0.232	0.425	0.486	0.0172

## Discussion

FEA is a computational tool that can be used to simulate the behavior of complex structures under different loading conditions [22]. FEA can be used to evaluate the structural performance of customized reconstruction plates and compare them with commercially available stock plates. FEA can also be used to assess the biomechanical responses of different plate designs, which can facilitate their structural performance optimization. In particular, this approach can help identify potential design flaws and improve the overall quality of reconstruction plates [20].

Biodegradable Mg-based alloys have been widely used for bone fixation in recent years. Magnesium alloys have been shown to be useful for fixing plates and screws in maxillofacial fixation [27–32]. The surgical removal of Ti fixation devices following adequate bone healing is generally advised to prevent complications in children, such as invagination of the fixation device into the cranial cavity, inhibition of bone growth, and tooth damage [33]. Although resorbable polymer devices have been developed to address these issues, their mechanical characteristics frequently limit their use in load-bearing applications [27]. The use of biodegradable Mg-alloy plate and screw systems can potentially address this challenge.

Magnesium-based alloys are promising materials for use in cardiovascular and orthopedic medical devices owing to their natural biodegradability through corrosion when placed in aqueous environments. Therefore, these materials can serve as temporary scaffolds within the body, rendering them suitable for treatment scenarios requiring temporary support structures to facilitate wound healing. The biodegradability of Mg-based materials is attributed to the high oxidation and corrosion rates of Mg [34, 35]. Resorbable plating systems have been successfully used to repair midface and mandibular fractures in the pediatric population [36].

Despite these promising results, the use of Mg-based alloys for therapeutic purposes is limited owing to their rapid corrosion under physiological conditions [28]. Recent studies demonstrated that the addition of alloying elements, such as Al, Mn, Ca, Zn, and rare-earth elements, can enhance the corrosion resistance of Mg alloys [37]. Furthermore, the effects of polymer coatings, synthetic aliphatic polyesters, and natural polymers on the corrosion and biological performance of Mg and its alloys have been discussed in the context of orthopedic applications [27, 38].

ZK60 exhibits suitable mechanical strength for maxillofacial surgery; however, it also exhibits a high absorption rate [31]. Various coating methods have been explored to decrease the absorption rate of Mg alloys during their biodegradation. A PLLA coating layer exhibited excellent stability when subjected to tensile force, which is a crucial quality for clinical applications. However, bending ZK60 damaged the PLLA coating layer owing to its brittle nature, indicating that the PLLA coating may not effectively reduce the ZK60 absorption rate for maxillofacial applications. WE43 exhibits high biocompatibility with minimal side effects but has low strength. In contrast, ZK60 generates a substantial amount of hydrogen gas but has high strength. Consequently, a selective approach is required for the application of these materials [32].

The analysis of the MPS of screw holes in this study showed that the Mg materials, as compared with those of the Ti materials in all groups excluding Group 1, exhibited a lower stress in the cortical bone and higher stress in the cancellous bone when subjected to a 500-N axial load. This highlights an advantage Mg has over Ti, that is, Mg can disperse stress from the cortical bone to the cancellous bone. However, a significant limitation of this study was that the plate thickness varied depending on the material properties. Because the elastic modulus of Mg is significantly lower than that of Ti, the Mg plates were designed to be thicker, resulting in a higher biomechanical strength of the implant system using Mg. Such configurations were widely used in existing studies. Resorbable plates, as compared with Ti equivalents, are less durable and bulkier for supporting a given load, rendering them less desirable for the treatment of comminuted or displaced midface fractures [6]. The plate thickness must be consistent for a more objective comparison, and future studies should consider this aspect.

Regarding the PVMS, the screw fracture risk was high in the 500-N axial and 250 N–60° loads when Mg materials were used. In contrast, the Ti material was predicted to be stable as the PVMS values were lower than the breaking strength under all loading conditions. Different plate sizes for Ti and Mg were used in this study to compensate for the strength differences between the two materials; however, the same screw size was used. Therefore, a higher predicted fracture rate for the Mg screw was expected. Sato et al. [39] reported that fixation screw failure is a potential complication in plate-reconstructed facial bones. Therefore, further studies are required to improve the strength of Mg screws.

The gap between the osteotomy surfaces was maintained in the model with Mg implants. However, the osteotomy surfaces experienced bone impaction in the model with Ti implants, resulting in gap closure. A suitable gap is required for osteosynthesis. A gap distance of approximately 0.5 mm was implemented in this study. The occurrence of gap closure with Ti implants indirectly suggests that their initial stability is lower than that of Mg implants. No significant variations in stability were observed due to plate shape or combination.

Masticatory forces range from 75 to 300 N [1, 3, 22]. Vertical loads of 100 and 500 N were applied to the premolar and molar regions of each model to simulate these forces. Additionally, lateral forces of 250 and 100 N at 60 and 90°, respectively, were applied. The upper part in the established model was limited to a total of six XYZ rotational movements and fixed parallel to the occlusal plane. The results may have varied depending on the fixed upward and downward positions. However, because the plates were compared under identical conditions, the proposed approach can be considered suitable for FEA-based comparisons. Nevertheless, it must be acknowledged that the bite force and loading point for patients may differ from those used in this experiment.

Overall, the use of Mg alloys as potential implant materials has garnered increasing interest owing to their biodegradability; however, their clinical use is impeded by their rapid corrosion during the absorption process. Various coating methods and alloying techniques using different materials have been explored. However, the corresponding biocompatibility and properties of the material must be comprehensively investigated. The initial fixation strengths of Mg and Ti alloys after implantation were examined in this study. However, only limited experimental conditions were considered, and further research is required to examine changes in fixation strength during Mg absorption. Such investigations can enable an unbiased assessment of the biocompatibility of Mg alloys as implant materials and their material stability during absorption.

## Conclusions

This study, based on FEA techniques, provides valuable insights into the biomechanical performance of biodegradable Mg alloys (WE43 and ZK60), as compared with Ti, for maxillofacial fixation. Magnesium, as compared with Ti, evenly distributed the stress between the bone screw and metal plate. However, challenges associated with the inadequate screw strength and rapid corrosion of Mg alloys should not be overlooked. To leverage the potential of Mg alloys for maxillofacial fixation, further research must be focused on enhancing the screw design and alloy properties to mitigate fracture risks. Moreover, the long-term corrosion behavior and its effect on fixation stability must be further explored.

This study contributes to the understanding of biodegradable Mg alloys as viable alternatives to Ti for maxillofacial fixation. Advancements in materials science and implant design can facilitate the identification of safer and more effective biodegradable solutions for craniofacial surgery.

## Data Availability

No datasets were generated or analysed during the current study.
